# How do Healthcare Workers ‘Do’ Guidelines? Exploring How Policy Decisions Impacted UK Healthcare Workers During the First Phase of the COVID-19 Pandemic

**DOI:** 10.1177/10497323211067772

**Published:** 2022-01-29

**Authors:** Caitlin Pilbeam, Sarah Tonkin-Crine, Anne-Marie Martindale, Paul Atkinson, Hayley Mableson, Suzannah Lant, Tom Solomon, Sally Sheard, Nina Gobat

**Affiliations:** 1 Nuffield Department of Primary Care Health Sciences, 6396 University of Oxford, Oxford, UK; 2 NIHR Health Protection Research Unit in Healthcare Associated Infections and Antimicrobial Resistance, University of Oxford, Oxford, UK; 3 Institute of Population Health, 4591 University of Liverpool, Liverpool, UK; 4 Institute of Infection, Veterinary and Ecological Sciences, 4591 University of Liverpool, Liverpool, UK

**Keywords:** COVID-19, healthcare workers, guidelines, policy, enactment, qualitative, pandemic response, interviews

## Abstract

We describe how COVID-19-related policy decisions and guidelines impacted healthcare workers (HCWs) during the UK’s first COVID-19 pandemic phase. Guidelines in healthcare aim to streamline processes, improve quality and manage risk. However, we argue that during this time the guidelines we studied often fell short of these goals in practice. We analysed 74 remote interviews with 14 UK HCWs over 6 months (February–August 2020). Reframing guidelines through Mol’s lens of ‘enactment’, we reveal embodied, relational and material impacts that some guidelines had for HCWs. Beyond guideline ‘adherence’, we show that enacting guidelines is an ongoing, complex process of negotiating and balancing multilevel tensions. Overall, guidelines: (1) were inconsistently communicated; (2) did not sufficiently accommodate contextual considerations; and (3) were at times in tension with HCWs’ values. Healthcare policymakers should produce more agile, acceptable guidelines that frontline HCWs can enact in ways which make sense and are effective in their contexts.

## Introduction: What do Guidelines Do?

The Institute of Medicine defines healthcare guidelines (henceforth ‘guidelines’) as ‘systematically developed statements to assist practitioner and patient decisions about appropriate healthcare for specific clinical circumstances’. (Woolf et al., 1999) These can include clinical practice, infection prevention and control, public health and occupational health and safety guidelines. Guidelines are evidence-based recommendations for best practice and also have an important role in medico-legal responsibility (Eccles et al., 2012). Synthesising from core UK administrative healthcare bodies, guidelines seek to accomplish three key things: to (1) streamline processes, (2) be a tool for quality improvement in patient care and (3) aid the management of risk (NICE, 2020; van der Weijden et al., 2010).

After the first confirmed COVID-19 case on 31 January 2020 (DHSC, 2020), the peak of the UK’s first phase spanned April to June 2020; as of 25 May 2021, the UK has experienced 4.46 million cases and 127,724 COVID-19-related deaths (PHE & NHSX, 2020). Here, we aim to understand the extent to which guidelines discussed by study participants, related to clinical care and managing health services, achieved these goals in practice during this phase. Further, we aim to describe the lived experiences of 14 healthcare workers (henceforth HCWs), whom we interviewed repeatedly over this time as they interacted with these guidelines and adaptations. Understanding impacts for HCWs of putting guidelines into practice during an emergency is integral to informing future emergency responses and guideline development, to produce more effective and appropriate guidelines.

Multiple official UK bodies disseminate guidelines to HCWs, including PHE (Public Health England), NHS England and Improvement, HSE (Health and Safety Executive), DHSC (Department of Health and Social Care), NICE (National Institute for Health and Care Excellence), individual Royal Colleges and individual Trusts. Guidelines are communicated to HCWs predominantly by cascading information top-down, most often digitally via official web-pages, emails, intranets, as well as in face-to-face briefings and laterally to each other. Keeping guidelines updated, aligned and informed by current evidence is an ongoing supporting task, especially pertinent in (global) public health emergencies, where the need for rapid guidelines is high and evidence may be rapidly changing (Vogel et al., 2019).

The COVID-19 pandemic has generated a vast informational landscape that HCWs must navigate, including more information than in non-pandemic contexts from multiple different sources and levels, local to international. Some argue, therefore, that there should be a focus on supporting HCWs, through ‘supportive conversations, clear guidance when recommendations exist, attempts to minimise misinformation and efforts to reduce anxiety’. (Adams & Walls, 2020:1440)

Day-to-day healthcare is standardised through guidelines’ recommendations, yet whilst guidelines themselves may be the same, implementation of these guidelines may vary greatly depending on context. Nevertheless, the importance of context in implementation may be overlooked by guideline developers (Greenhalgh et al., 2014), which may be reflected in the disappointingly modest impact these guidelines have on the quality of patient care (e.g. Fischer et al., 2016; Lugtenberg et al., 2009).

As some authors highlight, putting guidelines into practice is not straightforward, as ‘implementation requires a number of steps to translate the knowledge contained in guideline text into a computable format and to integrate the information into clinical workflow’. (Shiffman et al., 2004) Further, guidelines in practice are melded with shared and personal experience (Gabbay & Le May, 2016). Indeed, these processes do not follow the linear cause-and-effect model that evidence-based medicine often assumes. To produce desired outcomes, evidence-based medicine must be considered in context (Greenhalgh et al., 2014).

Much literature on guidelines focuses on evaluating specific recommendations, how to improve adherence to guidelines and overcoming barriers to HCWs following guidelines (Fischer et al., 2016; French et al., 2012; Lugtenberg et al., 2011). However, these perspectives are limited as they similarly imply a linear logic. They pay little attention to complexity or how guidelines become situated in lived context, and thereby do not account for the contextual realities of those doing the implementing. In this article, we therefore chose to employ the theoretical lens of enactment, to depart from notions of adherence and further consider how past experiences, present context and daily practice come together in ‘doing’ guidelines.

## Enactment: How do Healthcare Workers Do Guidelines?

We draw on Annemarie Mol’s notion of ‘enactment’ which foregrounds practicalities in context, attending to interactions through which different forms and realities emerge (Mol & Law, 2004:48–9). Enactment emerged from a theoretical tradition of Actor-Network-Theory and Science and Technology Studies. However, this term was specifically chosen as a concept with ‘less baggage’ than others of its ilk, like ‘performance’ and ‘practice’, whilst at the same time connecting these (Mol, 2002:33; Schwertl, 2016:168). Enactment foregrounds how interactions between human and non-human actors uniquely bring about or ‘create the situation and its entities or objects’. (Schwertl, 2016:169) Premised on multiplicity, therefore, enactment can describe the ‘multiple doings and beings of one disease’, object or role (Schwertl, 2016). The enactment, or ‘doing’, of guidelines is an ongoing and emergent process.

Here, we apply this to mean that the enactment of guidelines does not happen in one set way (e.g. ‘right’ or ‘wrong’ and ‘good’ or ‘bad’), in a context where multiple enactments and guidelines correspond. Whilst there is a ‘here and now, in which “doing” happens’, this doing is not necessarily ‘explained by what went before – there are patterns and routines, but there is also always the possibility of surprise’. (Martin et al., 2018) This lens also mitigates the danger of conceptualising the ‘impact’ of guidelines ‘on’ HCWs as a purely top-down, one-directional relationship. Enactment emphasises the complex and dynamic ways that HCWs take up multiple interrelating guidelines in context, against the more linear fashion that ‘following’ or ‘adhering’ to guidelines suggests.

Through the lens of enactment, doing guidelines occurs ‘within a fluid network of practices’, in relation to interacting ‘human and non-human actors’ (Schwertl, 2016) in a material environment. Adopting this lens therefore foregrounds relational and material aspects of how HCWs enact guidelines; this means HCWs doing something which necessarily involves interacting with others (e.g. patients/colleagues), both within and interacting with their physical environments (e.g. hospitals/resources). ‘Putting guidelines into practice’ for HCWs is thus contextually embedded in and unfolds through one’s own embodied performance, interpersonal relationships and material environments.

Enactment thus takes within its scope the wider implications of how putting these guidelines into practice produces and incorporates different activities, relationships and things. This offers a more nuanced understanding of context as peopled, multi-layered and interactive, rather than static and separate from individuals who act independently within an environment. The enactment of guidelines therefore unfolds at multiple interacting levels. As action is privileged over knowledge (Mol & Law, 2004: 47), a focus on this performativity helps us to look across different HCWs, their roles and relative seniority to learn something about the impact of guidelines across different contexts.

## Methods

We are an interdisciplinary group of researchers – including social scientists, historians and biomedical researchers – brought together by shared interests in how people and societies cope with infectious diseases. Reflecting on our positionality, we approached this research, from study design to data collection, analysis and interpretation by innovatively combining our expertise in social science methodologies, policy and clinical medicine in complementary ways. With an explicitly applied focus, we strengthened this article through multidisciplinary language and messages, aiming for the light-touch incorporation of theory (as above) and policy implications (below) to render our contribution legible and relevant to similarly diverse audiences.

### Study Design

In a remote longitudinal study, we used interview methods to capture HCWs’ experiences during the COVID-19 pandemic in the UK from February 2020 to February 2021. Here, we focus on the UK’s first phase and present findings from 74 interviews completed during this time. We interviewed a panel of 14 HCWs three to seven times over the course of 24 weeks from 26 February 2020 to 13 August 2020.

We contacted participants for interview every two to four weeks in the first instance, depending on their availability, and, after June, every six to eight weeks due to the UK’s decreased prevalence of COVID-19. Interviews were conducted by social scientists with prior training and experience in qualitative research methods and interviewing HCWs. We conducted all interviews via teleconferencing or telephone, and interviews usually lasted 30 minutes to one hour. We audio-recorded all interviews, and professional transcribers verbatim transcribed interviews which we identified as containing the richest data relevant to the aims and scope of the project (e.g. interviews were not transcribed where participants reported ‘no change’ or ‘nothing new’ in how they were managing and responding to the COVID-19 crisis, such as over the summer period when COVID-19 infection numbers were low). Interviewers kept brief fieldnotes of virtual encounters with participants to capture contextual features, themes and narratives. We also noted relevant news media items and official guidance documents.

Interviews were semi-structured around two key areas: (1) experience of clinical service adaptations and readiness and (2) perceptions of healthcare system resilience and response to COVID-19. We iteratively added to the topic guide to capture relevant themes. We explored adaptations to clinical practice and roles, management of patients with COVID-19 and provision of usual care, availability of resources (e.g. equipment, training, time, funding), perceptions of infection control and risk, collaborative working, patient experience, triage and end of life decisions and key challenges moving forwards.

### Participants and Recruitment

The interview panel consisted of nine women and five men based in primary, secondary or tertiary care services in England and Scotland during the study period. All participants were white European; given the disproportionate impact of the pandemic on Black, Asian and minority ethnic populations and HCWs (e.g. Hu, 2020), this sample is limited in that it cannot speak directly to these dynamics. We discussed and made an effort to recruit a more diverse and representative sample, although were unable to accomplish this more fully as sampling and recruitment was pragmatically driven during an exceptional period in the crisis phase of the pandemic, which we sought to capture; prioritising the longitudinal nature of the study, we chose not to reconfigure the set after we closed recruitment and recognise the limitation of the sample. Nevertheless, participants together offer a breadth of experiences, clinical disciplines, roles, responsibilities and seniority. These were particularly important aspects to capture given that the COVID-19 response involved redeploying HCWs to services outside their specialities, recalling retired HCWs, as well as HCWs disseminating, implementing and managing rapid changes to service delivery at multiple levels.

Recruitment was ongoing from early February to early May 2020, using a snowball method. We recruited pragmatically, initially through existing professional networks for speed and convenience, and then later disseminated social media adverts to recruit further and diversify the sample. Participants were informed about the aims and scope of the study, that is, to document the experiences of frontline HCWs and contribute to the pandemic response by (regularly) disseminating findings to policy and public audiences (e.g. COVID-19 Hot Potato). However, recruitment was limited by the very nature of conducting research during a pandemic with frontline responders; many HCWs we contacted did not feel they had the time and/or energy to dedicate to longitudinal interviews. The sample may therefore be biased towards those who felt more able to cope during the pandemic; however, the length of the interviewing window and ongoing nature of the pandemic meant that we were able to capture varying responses over time and build strong relationships with participants.

Three participants were general practitioners (henceforth GPs); three non-ITU nurses redeployed to ITUs (Intensive Therapy Units); two allied health professionals in secondary and tertiary care; two clinicians (doctors) based in accident and emergency departments; two clinicians based in obstetrics, gynaecology and neonatal services; one specialist consultant in tertiary care and one retired nurse who returned to the register during the pandemic. Two clinicians also held management and leadership roles as Clinical Directors, and a third was also a Clinical Lead overseeing a hospital medical department. More specific details are given throughout the results section below (e.g. roles and level of experience), where further identifying information has been removed to protect anonymity.

### Data Analysis

Data were analysed iteratively and interpretively in discussion with the wider research group, together inductively developing and refining our interpretation of findings throughout the process of data analysis (Silverman, 2011). We used Frank’s (1995:23) distinction between ‘thinking about’ and ‘thinking with’ stories to prioritise participants’ own experiences and accounts, as reflected in the presentation of data here. In practice during analysis, this involved holistic read-throughs of interview transcripts (Van Manen, 1997), as well as repeated listening to audio-recordings (Greenwood et al., 2017), identifying salient themes grounded in materials themselves; being familiar with the longitudinal narratives of each participant was important before breaking these data down.

**Figure 1.**
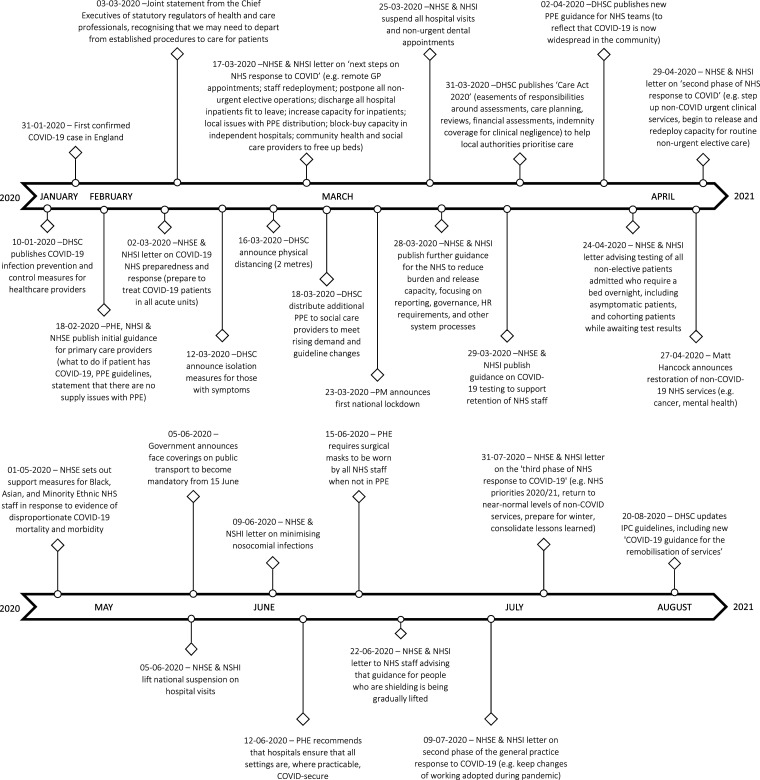
Timeline of UK COVID-19 National and Healthcare Guidelines.

Due to the length of data collection and large size of the dataset, we also kept a record throughout data collection of key aspects of individual interviews, highlighting particular processes, storylines and specific events that participants spoke about (e.g. news items, new guidelines and happenings at work). Throughout data collection and analysis, therefore, we contextualised data using a timeline of key policy decisions, guidance documents and media narratives on COVID-19 (see Figure 1) and compared and contrasted themes generated in our work with anticipated themes from findings in the emerging COVID-19 literature.

Interpretation for this article was developed with the wider research group, ‘zooming in and out’ (Nicolini, 2009) between granular data and overarching interpretation with regards to the theoretical lens to maintain closeness to participants’ accounts. All quotations used here were approved by participants, who also chose their level of identification (e.g. job role and workplace) (Mero-Jaffe, 2011).

### Ethical Considerations

Ethical approval for this study was given by the Medical Sciences Interdivisional Research Ethics Committee at the University of Oxford (R69302/RE001). Interviews were conducted only on digital platforms approved by the University of Oxford. Given the tensions and constraints of doing research with HCWs during a pandemic, we sought to be sensitive and flexible regarding participant recruitment and involvement. Informed consent was obtained by interviewers going through the consent form with participants via teleconferencing, participants completing the forms digitally and participants were assured at each successive interview that they could withdraw their participation at any time with no adverse consequences.

We were also aware of potentially difficult topics, experiences and conversations that interviews may bring up for HCWs. We assured participants that they could withdraw from the study at any time, pause interviews or decide not to discuss anything with which they felt uncomfortable. Despite recruitment challenges and time constraints, many participants expressed that they wished to contribute to research into the pandemic response. Participants also expressed how much they enjoyed interviews – similarly echoed in other interview studies conducted during the pandemic (e.g. Vindrola-Padros, et al., 2020) – as a space to process events and vent frustrations and uncertainties aspects with which researchers took care when addressing in interviews.

## Results

### Context: UK Guidelines

Participants discussed both national public health and professional healthcare guidelines, summarised in Figure 1 to contextualise findings. PPE guidelines and availability, accompanied by intense media coverage, changed many times throughout the response, so were not possible to capture fully.

We structured findings around three key themes that shaped and were shaped by guidelines and their enactment: (1) adaptations to HCWs’ practice in systems of interconnected services, roles and responsibilities; (2) implications of the realities of the pandemic itself; and (3) challenges of changes that may clash with pre-pandemic established standards of care. This section is broadly descriptive, in the vein of a realist telling (van Maanen, 1988), to give a sense of some of the day-to-day challenges and successes as experienced by HCW participants during the pandemic; in the discussion, we then bring the notion of enactment to bear more concretely on findings.

### Adaptations to Practice

‘The debacle about PPE is a real one. The changing scenario of “what PPE have we got today and what does it look like today, how do I use it” – if that had been uniform from the beginning, and uniform advice about PPE… [the emergency department] is a boundary, an interface between [different services], and they have all had different advice about PPE. And then us, and the hospital itself…it feels like sometimes there have been different standards. So I just feel that if we had had clear, uniform guidance and equipment for PPE it would have been a lot less stressful from the outset because we have changed it so many times. […] It is just that clarity of communication from the outset – even clarity of communication of “it’s not ideal, this is what we’re going to do now, but as soon as we have got this, we will do the next thing”. It’s just the uncertainty of PPE is a nightmare’.

A Clinical Lead in an Emergency Department (ED) describes above how PPE availability and guidelines were inconsistent and frequently changed, which she saw contribute to workloads and pressure. Combined with challenges of redistributing and keeping ED staff updated, she negotiates the interface with multiple other services where different HCWs may have had different PPE advice. These inconsistencies create difficulties and extra steps when transferring and caring for patients and related workflow issues. A Clinical Director said later in the COVID-19 response that he had ‘given up on planning, really’. Keeping up with changes and planning accordingly became impractical due to ongoing uncertainties and inconsistencies, which necessitated a reactive, rather than proactive, approach. Over time, this meant increased workloads, extra potentially unnecessary steps, that some HCWs felt may not have been required if guidelines and resource availability were communicated transparently, and the same information was relayed consistently.

Beyond PPE, there were further resource constraints highlighted by HCWs in leadership positions, particularly staff shortages, time constraints and insufficient digital and physical infrastructure to implement some changes rapidly (e.g. interoperable IT systems and sufficient space on premises). Large-scale changes to workforce and ways of working may understandably generate additional workloads, requiring new systems and standard operating procedures, for example. Participants often saw building new infrastructure as rapid and welcome progress, yet many also felt that additional burdens were partly avoidable through more engagement when developing guidelines with the HCWs who would implement them. Almost all HCWs interviewed expressed a pragmatic attitude of finding ways to make things work in imperfect conditions, encompassed by the phrase ‘just get on with it’, yet the uncertainty and sheer volume of changes and new information in the pandemic could overwhelm HCWs. Successes and challenges often came hand-in-hand.

Two GPs both describe how moving to remote consultation entailed overhauling IT systems and software, to accommodate guidelines to reduce face-to-face patient contact. This enabled them to share a virtual front desk with neighbouring practices to better manage patients, staff shortages and workflow issues. Arthur describes how online consultations increased access for some patients, yet also contributed to patients expecting and GPs feeling that they had to offer 24/7 healthcare, putting additional strain on GPs. GPs felt work was further duplicated when centralised letters (advising vulnerable patients to shield) simultaneously missed some patients and targeted others inappropriately, and patient referrals from primary to secondary care were shut down. Whilst blanket policies might streamline processes for one part of the health and social care system, this may increase work for HCWs elsewhere, illustrating the non-linear effects of enacting guidelines.

Workflows were also disrupted by changes to material environments and daily caring routines. For redeployed staff in particular, this meant moving through often unfamiliar spaces and specialities they must learn to navigate, adding to their workload and decreasing efficiency. A respiratory nurse who was redeployed to ITU said,

‘You don’t know what you’re in for… we’re split across three floors so you go to the sixth floor one night or day, third floor another, first floor, and then you’re back on three and then you’re back on six. And the layout in [this hospital] is awful, so trying to get familiar is difficult […] Once you start to know a place, you get taken off and then you’ve got to try and work it all out again’.

He describes the practical difficulties of joining a new team in a new environment in the midst of the pandemic, and how daily tasks were complicated by not knowing where things were or who to go to in his new social and physical environment. Reflecting on how he struggled with these smaller yet consistent challenges during redeployment, he expressed relief when able to return to his original role: ‘it is just the familiarity which makes it a lot better’.

Clinical spaces split into COVID-19 and non-COVID-19 zones also directly impacted how HCWs moved through hospitals, for example, necessitating different routes depending on the HCWs’ status as ‘clean’ or not, and barring entrance for some HCWs to some spaces altogether. One participant notes: ‘You used to be able to sneak through corridors left, right and centre – it is a big hospital on 11 floors, and you could zoom through, and that is all gone’.

Many HCWs had found their own ways of moving that suited them, but during the pandemic their movement became more standardised, if not optimised, and greater emphasis was placed on compliance. Whilst this was not problematic for some, HCWs described instances where these changes had wider and potentially unanticipated impacts, such as on wellness.

‘Another thing with wellbeing […] The canteen and the shop are both going to be clean [non-COVID zones], so unless you’re working in an elective or planned admission zone you basically have no way of going to get food or drink’.

Others highlighted that this similarly meant that those who were perhaps most in need of support were unable to use some wellness resources, such as ‘wobble rooms’. Putting up barriers that HCWs must (often physically) navigate renders them less able to make their environments work for them, and thus have implications for their wellness and strategies for resilience. A specialist nurse redeployed to ITU emphasises that the way these changes were first implemented in her locale involved no consultation with the staff who ultimately navigated them and resulted with her and colleagues not even being able to make a cup of tea.

‘They bring this whole zoning thing in, and don’t speak to a nurse about it that would be using it… So our office is right next to the ITU, which is obviously the most “COVID” zone in the whole thing, which meant that we were in a COVID zone but we can’t make a cup of tea because the kitchen is over there in a clean zone – it all got so complicated to try and work out. We couldn’t access our kitchen, our fridge, our corridor […] I love the idea but it’s desperately hard to implement’.

She later confirmed that the hospital quickly amended zoning once highlighted, yet these concerns nevertheless speak to how guidelines tangibly impact HCWs’ bodies – particularly zoning, PPE requirements and emergency staffing. Another redeployed nurse describes how ITU staff where he worked were unable to leave the ITU space very often, as this would require doffing and disposing of PPE – which took time and was then nationally short and non-reusable. Unable to go to the bathroom or for water breaks for many hours, he describes a careful balancing act that he said left HCWs feeling unwell.

‘Having to put [PPE] on, especially when the weather’s been warm recently, that’s hard because there’s no air-conditioning, and you might as well be wearing winter gear […] I think that has been the hardest bit for everyone. The dehydration and the masks. It’s like you feel heavy, you know? And you’re there all day. Even if you do get a break, it’s a twelve-hour day, so yeah, you can imagine how rubbish that is. And people’s faces – they’ve been giving us cream […] Moisturiser, really, just for like our hands and our faces, just to stop it from drying and people getting pressure sores on their nose. People are getting sores here, behind their ears, from where the masks or the goggles sit all day, and yeah – it’s not comfortable. It’s not comfortable at all’.

Enacting and layering multiple guidelines in context comes together in daily practice to culminate in severe workflow challenges that HCWs must consistently strive to navigate. Changes to workload and workflow both have implications for efficiency and add burden to HCWs. Some HCWs describe having to find their own solutions and workarounds, such as being strategic about the order in which they saw patients and moved through clinical spaces, from non-COVID-19 to COVID-19 zones. In many cases, this meant that some HCWs were not able to see patients at all. A speech and language therapist in a hospital said,

‘We’re trying to not overuse PPE and trying to make every contact count – such a slogan – but I guess trying to be smarter about the way we see people. Yeah, it feels really weird…if we’re not seeing [a patient], I’m always questioning myself: “Am I not seeing this person because I’m trying not to waste PPE?”’

She openly discusses the tensions and struggle she felt when multiple guidelines collided in context, and the impact this had on the care she was able to give patients. Other HCWs described similar issues.

The introduction of new guidelines and recommendations during the pandemic often required rapid activation and reorganisation. Enacting guidelines day-to-day means negotiating between and layering multiple smaller and larger alterations that together stack up to create diverse and interacting effects. For HCWs in this study, enacting guidelines meant tangible, impactful material changes. HCWs at all levels expressed that these changes, although understandable, took increased time, human and material resources to put in place and in many cases increased workloads. Whilst this was expected to some extent, HCWs found that inconsistencies in guideline communication, with structural and resource constraints, contributed to potentially unnecessary additional burdens, such as barriers to planning and preparedness, caregiving and self-care.

### Implications of the COVID-19 Pandemic

‘We were essentially trying to almost work in war-like conditions, where you’re trying to save lives. But it is quite hard when you’re used to giving really good care to a very high standard, with all that holistic care, whereas obviously the holistic model has gone now. […] The holistic model is so what I’m about, you know. I think it just felt a double-edged kind of trickiness to be moving to this task-orientated care’.

A specialist nurse redeployed to ITU describes above the absolute priority during the UK’s pandemic peak to save lives. She highlights, however, how she felt that this was often at the expense of other important aspects of care. Her account underlines how these parts of caring are important not only for patients, but also for HCWs who, at peak, rarely saw the patients they were caring for recover. She explains how this single-minded focus on saving lives to the exclusion of more holistic priorities was a consistent frustration that she felt made her time in ITU even more difficult.

‘I love the simple stuff, you know, it’s always the detail that matters to me. That’s why I need to get out of ITU – it’s not the life-saving blood pressure stuff, it’s the, you know, making sure… the patient’s cared for and washed and clean and looking like your own relative, how you’d want them to look […] and I know from patients how important it is to feel how, you know, when you’re sitting in a sweaty bed for 12 hours you want someone to come and make you feel lovely again’.

This frustration with unaligned values was similarly felt outside ITU by HCWs whose specialities were not very highly prioritised. A registered dietician based in a hospital describes that, in her workplace, the combination of requirements for PPE and equipment shortages contributed to prioritising PPE for HCWs deemed to give ‘essential’ care. This meant that she became unable to see patients face-to-face for a time, and an alternative protocol for renal patients was implemented, giving them only supplement drinks (containing energy and protein), without her team being consulted.

‘It was completely the opposite of what I would normally do. […] There are so many [other] things that you can do. Even counselling and, you know, speaking to them – it makes such a difference – or looking at them. I can tell if someone is malnourished by looking at them, and I didn’t have all of that. I was trying to get the nurses to fill in the food charts’.

She expresses her frustration with how guidelines were enacted, which meant that the quality of care was not prioritised, and resulted in the adoption of an alternative that she, as a specialist, considers a last resort. She acknowledges that reducing face-to-face care reduced potential for transmitting COVID-19 and preserved PPE at the time. However, she worries that below-average care will negatively impact patients, especially as HCWs caring for those patients did not have specialist training in dietetics. Nurses were not on the lookout for dietary issues, and she describes occasions when patients were missed who were not eating at all. Whilst the registered dietician was able to set up to work remotely, calling inpatients and outpatients for discussions, the way that guidelines coincided and were enacted in context meant that the level of care she wanted to provide was unavailable to patients.

A specialist nurse redeployed to ITU also describes feeling that she was not giving the standard of care she would usually, and how this ‘just felt wrong’. She emphasises that whilst compromises were necessary, these trade-offs sometimes seemed to come at the expense of the most humane aspects of care. She recounts a particular event with a patient:

‘He was diabetic, and he came back in my head because I feel I did not provide the care I would have in in a normal situation. So there is a team of professionals who come to the unit to turn patients, they’re called the Proning Team. They came to turn this patient and they found that he was soiled, so they asked me if I wanted to clean him before they turned him – and I said that I would rather do it once he had been turned so I could have plenty of time to do it properly. However, I felt pressurised to do so before they turned the patient. So there I was, cleaning this patient who was prone, so with his tummy on the bed. He was completely soiled, and it was a group of seven people looking at me washing his bum. So there was not much dignity at all in that situation, and it wouldn’t have happened in a normal situation. […] I totally understand that the priority was to turn this patient and make sure that his breathing was okay, and yeah, I totally understand that they have to turn a lot of patients and they cannot… I totally understand what the priorities are but – yeah, still’.

She illustrates the practical struggle it was to maintain important aspects of care whilst many priorities and ways of working were changing. ITU nurses, who usually care for one dedicated patient, had to care for multiple patients in many cases due to increased ITU capacity but insufficient staffing. She says she saw other nurses managing similar situations and when the above event happened: ‘I could not even draw the curtain back because I was keeping an eye on another patient who was intubated’.

These accounts highlight an important clash of priorities. HCWs must at once save lives and avoid death, whilst simultaneously being aware that saving lives should not practically be at the expense of what makes care ‘good’. A respiratory nurse redeployed to ITU contrasts current with the pre-COVID-19 guideline landscape.

‘In any normal situation, […] you’ve got the NICE guidelines. You’ve got the NMC [Nursing and Midwifery Council] Code of Conduct. You’ve got all these things that you have to adhere to and follow, so if you ever slip up on any of those and it’s questioned, then you would be brought up for it. And so during this time [the pandemic], I’m pretty sure that most people didn’t follow the [pre-COVID-19] guidelines. It was just: “at all costs to try and keep these people alive if you can”, and so, yeah – it felt how you could imagine being in a warzone. Just get on with it. Don’t worry about these guidelines or guidance that otherwise exist. You’ve just got to try and keep these people alive’.

The priorities of care during a pandemic cannot be divorced from the context in which they are embedded. These priorities are enacted through guidelines and impact real-world lives, not only patients and their families but also for the HCWs giving that care. A neonatal registrar in Scotland drives this point home, saying that sometimes ‘humanity needs to take precedence’. In a relatively low-risk speciality for COVID-19, she discusses mask-wearing and other guidelines early in the pandemic, before mask-wearing was compulsory:

‘It is not practical all the time – it’s not practical during patient interaction. […] If we’re just updating them on the ward round, obviously we would be at least a metre away from them and all of that. But if we were in a situation with them where we were conveying bad news or something like that, you’re with them. I don’t know anyone who would socially distance when they’re telling someone something really devastating’*.*

She highlights how compromises made when caregiving and enacting guidelines are contextual. Acknowledging that compromises are necessary, HCWs’ accounts highlight how less prioritised aspects of care are nevertheless important throughout, even in crisis situations. Beyond the challenges and tensions that HCWs navigated, when balancing competing priorities at peak, a specialist nurse redeployed to ITU describes how the way guidelines are enacted also has longer-term impacts.

‘In those early days when I kept saying: “this feels really unsafe, I’m worried about standards of care, I don’t feel I’m giving good care”. I was kind of reassured that it was all just about saving lives, that we’d be in a bit of warzone – which an element of that I get – but clearly I was right. In the fact that people are very much looking back at that and saying: “Why has this happened and why has that happened?” […] It’s always going to get looked at, you know, people are then excited to be alive and thinking: “Why have I got this pressure sore, why have I got this infection, that’s now the problem that wasn’t the original problem?” And obviously they’re really sick and they would maybe have got those infections anyway, but it just feels like another kick in the teeth’.

The immediacy of saving someone’s life often overshadows the aftermath of what patients, their families and HCWs may subsequently have to live with. HCWs in this study expressed that they felt frustrated and uncomfortable that COVID-19 guidelines did not match up to their own values of what good care should look like. Diverse aspects of care beyond life-saving are therefore nonetheless important in emergencies, temporary by nature and have potentially long-lasting impacts. This clash of values in combination with different situations meant that HCWs were at times unable to provide high-quality care and found it difficult to reconcile the contextual balancing act of compromises, priorities, risks and consequences that they had to daily negotiate.

### Challenges of Change

‘[There was] a period of a number of weeks where things [guidelines] changed almost daily, and in fact at one point the recommendations changed twice in one day, which was interesting. Things floated around, it felt like a lot of rumours: “We’ve heard this”. Or, “We’re being told this”. Sometimes conflicting information from some senior people on the ward, which was quite a challenge […] And we worry as well that if something suddenly takes 180° turn, whether and what has been the risk to us and patients when we were doing it that way before – and it’s now changed’.

A hospital-based speech and language therapist underlines above the tensions that become foregrounded when guidelines change, particularly regarding the perception of risk. She worries that a significant change in recommendations indicates that HCWs were acting riskily prior to that change. Describing ‘rumours’, she expresses that she that does not quite know who or what to believe; frequent changes begin to erode her and others’ trust in guidelines. Practical difficulties of keeping up with changes and resource shortages combine to further cloud decision-making about how to minimise risk. As she describes when deciding how to use PPE during her patient contacts:

‘You’re caught because you don’t want to expose yourself, and then potentially expose your patients or your loved ones. But you also don’t want to be that person who wasted the PPE because you were worried about yourself and maybe somebody else could have had it’.

These tensions are underpinned by the lack of transparency around how guidelines are developed, what guidelines are based upon and thus how they should inform best practice and risk management. In the context of resource shortages, HCWs express worries that guidelines change for reasons other than rigorous scientific evidence. The speech and language therapist said,

‘If I’m honest, I think there’s a little bit of mistrust that maybe – although we probably couldn’t put our finger on who – I think there’s a sense of wondering how much of the guidance is influenced by things like the supply chain and decisions that have to be made. So perhaps there’s a little bit of cynicism’.

Other HCWs echo these stirrings of mistrust, highlighting concerns that the priorities of those setting the guidelines may not align with the values and priorities of HCWs caring for patients. A specialist nurse redeployed to ITU said,

‘It’s not that I don’t trust [guidelines], but, being in a scientific profession, I think we want whatever decision is taken to be backed up by, you know, “We are doing this because it’s safer. It’s safest for the staff, it’s safest for the patients and this is the evidence”, rather than, “We are doing this”, without giving an explanation, because you might think: Oh, you are doing this because it’s economically more viable’.

These wider contextual considerations have important impacts on how guidelines are enacted, as Bonnie and others experienced. HCWs describe feeling ‘torn’ between managing one’s own risk and others’, combined with not knowing how to quantify that risk, what risks are necessary to take in order to provide ‘essential’ care and wanting to give good care. A hospital-based registered dietician illustrates this daily balancing act when emphasising how ‘really, really, very passionate’ she is about her patients, ‘but it’s just really hard to know what is ‘essential’ and what is not ‘essential’, it’s not black and white’ When resource shortages eased and guidelines for her procedures did not require full PPE, she was offered the opportunity to see inpatients face-to-face. Poignantly, she puts it: ‘I was struggling between doing my job and not doing something stupid’.

The registered dietician highlights that these tensions were exacerbated by not trusting that what was recommended in guidelines was sufficient to minimise her and others’ risk, echoing other HCWs’ concerns. She describes deliberately going beyond what guidelines recommended as part of how she enacted them.

‘I was so careful in the Department, I would wear a mask going around [before mask-wearing was mandatory everywhere], I wouldn’t go and see any patients, I was waiting for other people to finish with preparing their meals and then I would have my lunch after, in my little corner, I would disinfect everything I touched, but then people will still sort of come closer to me, to an extent, they were like: “It’s the protocol, you’re fine” But I just don’t know if that protocol is based on evidence or if it just suits the service, in a way? […] I didn’t really trust that what was recommended was enough’.

A specialist nurse redeployed to ITU recalls a particular instance that illustrates how the convergence of multiple guidelines during an emergency necessitated the tolerance not only of different standards of care but also, therefore, different kinds of risk for patients.

*‘*A consultant […] is putting in a tracheostomy at the bedside because he can’t do it in surgery, because the theatres are closed and for the real emergency cases only that were still coming in – and then he nicks the artery, this lady bleeds out. […] But sort of: “It’s just COVID”. That’s kind of the thing that in any other circumstance they would be like: “Why was it done at the bedside? Was the proper procedure done?” All these things and that person would have to explain themselves, whereas during this time they didn’t. […] But it was an accident and these things happen and usually, when you sign a consent form, they say one in 10,000 this will happen and probably she was that one’*.*

This nurse previously discussed how it was not always possible to enact pre-COVID-19 guidelines, developed with the specific aim to minimise such risks. New guidelines therefore sometimes precluded different courses of action, so that, in some instances, it seems that specific risks were unavoidable. From these accounts, it seems that certain kinds of risk were deemed acceptable under the circumstances, especially when there were no alternatives. Another nurse describes similar issues whilst redeployed to ITU, where her concerns were minimised at the time by superiors.

‘We had quite a lot of infection rates and pressure sores where obviously we were not doing the one-to-one [nurse-to-patient care], we were doing one-to-four and one-to-six. And I told you about my anxieties where we weren’t turning people as frequently because there wasn’t anyone there and we weren’t able to. The infection control just wasn’t there because we didn’t have the equipment. Each bed space we’d share […] Now they’re looking at all of that and […] they’ve said they’re going to retrain us on how to do aseptic technique! That was never the issue – that we didn’t know how to do it. It was more that we couldn’t do it’.

The COVID-19 emergency legitimised the prioritisation of certain kinds of risk over others. HCWs in this study expressed their concerns about risks taken, which arose through the interplay of new guidelines, lack of transparency around guideline development, structural and resource constraints and the pandemic context. Whilst emergency status may change the way compromises and trade-offs are considered, and decisions made, emergency conditions and measures cannot persist indefinitely. These circumstances meant that HCWs felt unsure about how to best manage risk and consistently had to balance compromises when making potentially difficult decisions.

## Discussion

In this article, we aimed to evaluate guidelines in practice and describe participants’ lived experience during the UK’s first COVID-19 pandemic phase, to better understand the impact of these guidelines for HCWs. Our findings therefore explored what doing guidelines in daily practice means, and what is at stake, for different HCWs. Results show the dynamic interaction of guidelines with their context: enacting guidelines goes beyond HCWs doing the actions specifically mentioned in a specific guideline. The enactment of a guideline, and therefore its impact, is thus often far broader than its content.

We identified three crosscutting themes: guidelines were inconsistently communicated, particularly in terms of being frequently changed, potentially conflicting and lacking transparency; guidelines were embedded in structural and resource constraints, which many guidelines did not accommodate; and guidelines were at times in tension with HCWs’ values and pre-pandemic established standards of care, demonstrating a clash of priorities.

These overarching characteristics contributed to HCWs enacting guidelines that did not always achieve their goals of streamlining processes, improving quality of care and managing risk. Practice adaptations brought successes and challenges, but often augmented workloads and challenges that participants felt may have been avoidable. Guidelines impacted care, but participants were often constrained in caregiving to the standard they felt necessary. Moreover, enacting guidelines foregrounded conflicts that further contributed to participants’ uncertainty around how best to manage risk.

### Guideline Consistency and Transparency

Participants’ accounts broadly echo other HCWs’ reception of UK COVID-19 guidelines, which can be illustratively but not comprehensively cited here. Policy, social and news media and HCW interview analyses in May (Vindrola-Padros, Andrews, et al., 2020b:6) found that inadequate training for redeployment, ‘rapidly changing guidelines, limited PPE and lack of routine testing created anxiety and distress [for HCWs] and had tangible impacts on efforts to maintain a sustainable workforce’. Insufficiencies and confusion around healthcare guidelines have been compounded by poor communication from central authoritie and contradictory guidelines introduced by different authorities (e.g. Cappuccio, 2020; Horton, 2020).

The role of scientific evidence in guideline development has also been repeatedly questioned, the scientific community pushing back on multiple recommendations (e.g. Shell, 2020). Many participants and other UK HCWs highlighted mistrusting guidelines that frequently changed, often independently of the scientific evidence upon which guidelines should be based (Godlee, 2020). Citing potential economic or political drivers, HCWs here and elsewhere call for transparency about the reasons behind changing guidelines, as these frequent changes, conflicts and inconsistencies gave rise to logistical challenges, increased uncertainty around how to engage in best practice, and worries of enhanced risk (Alwan et al., 2020).

### Contextual Considerations

Contextual factors, including structural and resource constraints, had a significant role in shaping how HCWs could enact guidelines in different locales. HCWs highlighted inadequacies in systems and processes, technological and digital infrastructure, physical premises and space, staffing, time, equipment, intra- and inter-department relationships. Rather than framing contextual factors as barriers to guideline implementation, enactment foregrounds how HCWs negotiated these as dynamic and interacting lived realities. This foregrounded mismatches between guidelines and local contexts.

Whilst enactment is an ongoing process, guidelines focus on discrete outcomes (Gabbay & Le May, 2016; Greenhalgh et al., 2014). Those developing and disseminating COVID-19 guidelines adopted a necessarily pragmatic approach to save lives despite limitations, yet were often seen to fail to acknowledge or accommodate contextual constraints (Greenhalgh, 2020). HCWs similarly commented that top-down approaches to guideline development and dissemination did not sufficiently engage frontline HCWs and their contextual realities as being part of wider systems and relationships (Vera et al., 2020), thus compounding implementation challenges. This further contributed to frustration and HCWs adopting a reactive approach to COVID-19 guideline enactment. Eroding trust and patience, this also contributed to retarding the rapidity, efficiency and preparedness of responses (Forman et al., 2020; Ng et al., 2020).

### Clashing Priorities

Guidelines themselves may become inscribed with the goals, assumptions and value judgements of those designing them; for HCWs, enacting guidelines by extension means enacting these priorities as well. A study by Grol et al. (1998) found that clinicians are more likely to implement well-defined clinical practice guidelines that are compatible with their current values. Participants here described how implicit priorities in guidelines, focusing on life-saving ‘essential’ care, seemed to clash with their own sense of caregiving as a HCW, including important ‘human’ aspects. Impacts for HCWs thus also accrued along the lines of what constitutes ‘essential’ care, and who is able to give it.

Participants clearly expressed an appreciation of the extraordinary circumstances of COVID-19, but many nevertheless struggled with reconciling trade-offs and compromises that they felt were sometimes contradictory to pre-COVID-19 guidelines and standards of care. Such contradictions may contribute to HCWs fearing that actions excused during the pandemic may be later judged unfavourably, for example, beyond medico-legal responsibility, contributing to moral injury (Greenberg et al., 2020). Indeed, much literature highlights the importance of protecting HCWs’ wellbeing in pandemic situations (e.g. Vera et al., 2020).

### Enacting Guidelines in Context and Impacts for HCWs

When putting guidelines into practice, Gabbay and Le May (2016:402) highlight the interlacing dynamics of guidelines with HCWs’ own experiences, knowledge and values, that is, ‘mindlines’. Acquired over a lifetime, mindlines incorporate HCWs’ training, interactions, local understandings and learning, to handle ‘many conflicting demands and a host of other influences’. The authors emphasise that ‘mindlines are much more flexible, malleable and complex than guidelines could ever be’. They argue that guidelines themselves are not best suited to ‘coping with clinicians’ many roles and functions’. Rather, HCWs must rely on the complementarity of guidelines and mindlines.

However, for novel diseases emergencies, HCWs have limited past experience to draw upon. Moreover, COVID-19 guidelines often contradicted accumulated expertise. Participants emphasised caring activities, relationships, bodily sensations and dynamic material environments, not the efficient processes, quality improvement and abstract conceptualisations of risk found in guidelines. Abstract, outcomes-oriented clinical guidelines thus had tangible impacts for HCWs.

For example, the directive to wear a mask or PPE may not be as simple as putting on these items at the beginning of the day and taking them off once one’s shift is over. For different HCWs, enacting this guideline entails wide-ranging processes and consequences, involving diverse experiences and informed by what is at stake.

Actually wearing a mask *means* that a neonatal registrar has to try to carry out normal care whilst communicating through a mask, and where she was unable to do so she removed her mask to calm a hysterical mother. Having to wear a mask *means* that a registered dietician cannot see her patients because there is no mask for her to wear, as she is not considered to give ‘essential’ care. Having to wear a mask *means* that a speech and language therapist worries about whether the care that she provides is ‘essential’ and if she is taking a mask away from someone else who would put it to better use. Having to wear PPE *means* that a redeployed specialist nurse ends his ITU shift dehydrated because he cannot often drink throughout the 12-hour shift. Having to wear PPE *means* that, whilst there are shortages of staff and equipment, another redeployed specialist nurse is one of few able to provide ITU care, so she is asked to care for up to six individuals at one time, which she does not feel is safe for her patients or herself.

These examples illustrate what is at stake for different HCWs when enacting COVID-19-related guidelines in their local contexts. We thus show how guidelines have impacted HCWs’ own bodies, and how they move through material environments; their relationships with themselves, colleagues, patients, authorities, others and things; and how they balance priorities and risks in patient care. Are these wide-ranging impacts to be expected or excused in pandemic situations? Whilst crises legitimise certain risks and compromises, emergencies cannot last indefinitely and impacts for HCWs and others may be far longer-term.

## Conclusions and Implications

The success of guidelines depends on their implementation and impact. We reflected on the less-reported embodied, relational and material impacts of enacting guidelines and their influence on caregiving. We explored these aspects in relation to core aims for guidelines – to streamline processes, improve quality and manage risk – showing that in practice guidelines often fell short of meeting these aims. Enacting guidelines is a complex process of navigating multilevel tensions, relationships and priorities, as well as material and informational environments. Guidelines are therefore not just about a single recommendation influencing individual HCWs’ behaviour. Rather, multiple guidelines interact in context and clearly impact the bodies, relationships and environments in and through which HCWs’ daily practices unfold.

These considerations are central to guideline development and dissemination in emergencies. Practically, our findings highlight the need for more diverse input into pandemic planning in healthcare facilities, to accommodate variation and nuances and render these processes less top-down. This should include engaging HCWs themselves, architects, engineers and social sciences. COVID-19-related guidelines required wholesale overhauls of material environments and ways of working and relating; these changes were not always supported by the necessary infrastructure, resources, communication channels or strategic planning. Pandemic planning should similarly address these needs for infrastructure support, (re)training and accounting for HCWs as people first.

Aspirationally, future health emergency responses should consider the ongoing impacts of mismatches between guidelines and context, and incompatibilities between HCWs’ values and the priorities implicit in guidelines. Guidelines and policies are often not considered at systems level; however, implementation always unfolds within relational systems of interconnected services, roles and responsibilities. Whilst the realities of a pandemic (e.g. novel pathogen and new knowledge continually emerging) contribute to rapid change, lack of consistency in guidelines especially across services led to loss of trust and questions about whether this ‘new knowledge’ is indeed driving guidelines. HCWs may be better able to tolerate changes if guidelines better accommodate and reflect their values, contextual challenges and realities.

Finally, whilst HCWs maintained a pragmatic attitude of ‘just getting on with it’, this attitude should not be taken for granted. To do so could mask structural inadequacies and contextual constraints that need to be improved. Further, there is a need for current and future research to be conducted with Black, Asian and minority ethnic respondents in the face of health crises. These experiences, for COVID-19 and potentially future pandemics, are likely to highlight important nuances and differences which would further support the development of more appropriate and acceptable guidelines for a more inclusive range of HCWs. These are aspects to which our own data could not speak, due to the unrepresentative nature of our sample. Especially given the disproportionate impact on these populations, we strongly suggest this be a future research priority in studies of and recommendations for guideline development and implementation, and pandemic preparedness. Overall, there should therefore be a greater appreciation of what doing guidelines means in practice for different HCWs on the ground, and what is at stake for them.
